# Reply to ‘placoderms and the evolutionary origin of teeth’: Burrow *et al.* (2016)

**DOI:** 10.1098/rsbl.2016.0526

**Published:** 2016-09

**Authors:** Martin Rücklin, Philip C. J. Donoghue

**Affiliations:** 1 Naturalis Biodiversity Center, Postbus 9517, 2300 RA Leiden, The Netherlands; 2 School of Earth Sciences, University of Bristol, Bristol BS8 1TQ, UK

Establishing the evolutionary origins of teeth is difficult not least since researchers disagree on whether or not the earliest jawed vertebrates, the extinct placoderms, possessed teeth. We recently showed that a gnathal plate from the acanthothoracid *Romundina stellina* comprises marginally added enameloid-capped tubercles. Burrow *et al*. [1] present concerns over the origin of our material, both taxonomic and topological, as well as the histological interpretation of the component tissues; none of their points are sustainable.

The skeletal plate that we interpreted as a gnathal of *Romundina stellina* (figure 1*a–d*) originated from the unsorted and unstudied personal collections of Tor Ørvig, derived from the same samples as the holotype [2]. Evidence of their attribution to *Romundina* is based on the shape and organization of tubercles, semidentine composition (placoderm-diagnostic tissue) and their co-association with *Romundina*, the only placoderm described from the locality. Unusually for placoderms, the dermal tubercles in *R. stellina* have enameloid caps [3], as do the morphologically distinct tubercles of the oral plate [4]. Support is found in another specimen previously attributed to *Romundina* [5] with a single rostral pair of gnathals, the structure of which has not been described. Its precise taxonomy is moot but irrelevant here since the articulated specimen is an acanthothoracid closely related to, if not, *R. stellina*.

**Figure 1.**
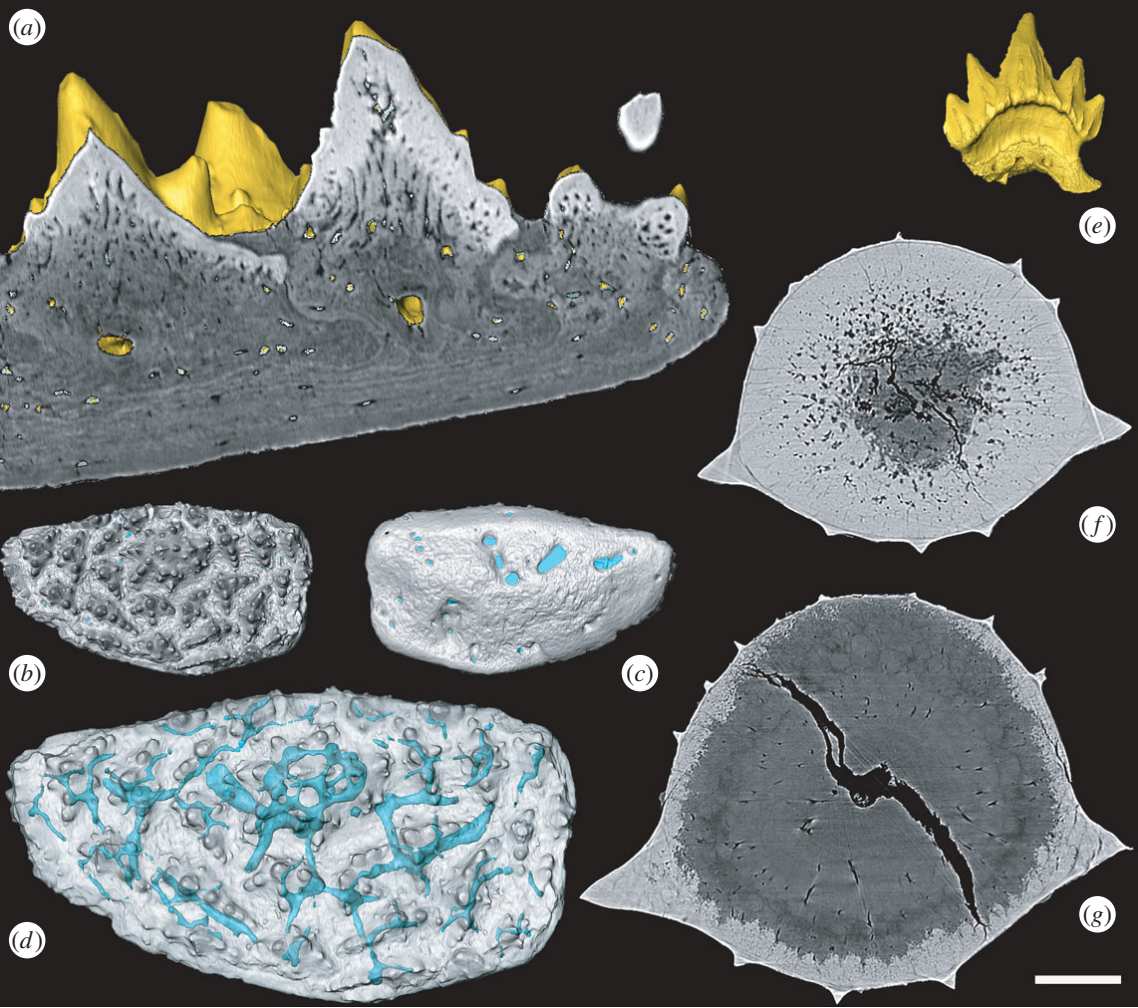
Surface rendering and virtual thin-section of *Romundina* supragnathal (NRM-PZ P.15956, *a*–*d*) and *Scyliorhinus canicula* (BRSUG 29402) teeth (*e–g*). *Romundina* pulp cavities in transverse section (*a*) and vascular system in oral (*b*), dorsal (*c*) and oral transparent view (*d*). *Scyliorhinus* tooth (*e*) enameloid layer with radial structures (*f*,*g*). Scale bar represents 50 µm in (*a*), 417 µm in (*b*,*c*), 240 µm (*d*), 480 µm (*e*), 37 µm (*f*) and 45 µm (*g*).

The skeletal plate we described (figure 1*a–d*) is compatible with the articulated gnathal plates, comprised from approximately concentrically arranged rows of branched tubercles [4, fig. 1(*a–d*)]. Differences in overall outline and size reflect growth; the isolated oral plate is equivalent to the inner core of the articulated plate, lacking larger, later superimposed tubercles. We demonstrated that the oral plate grew by marginal addition of tubercles [4, fig. 1(*a*)], substantiating previous suggestions [5–7]. Burrow and colleagues contend that the gnathals of coeval arthrodires have concave cancellous gnathal bases and possess tubercles that increase in size through ontogeny, but we did not attempt to interpret the toothplate as belonging to an arthrodire. Acanthothoracids, like ptyctodontids, are distant relatives of arthrodires and the oral and aboral morphology of their gnathals are concomitantly distinct, reflecting differences in the surface they attach to [6]. In *R. stellina*, the supragnathal is associated with the flat surface of the ethmoid region, bordering the premedian plate anteriorly. In the premedian plate of an adult *Romundina*, perichondral and dermal bone are indistinguishable [8]. Both the articulated and isolated gnathals have central symmetrical tubercles surrounded by asymmetrically branched tubercles that are distinct from dermal tubercle morphologies (figure 1*b*) that typically possess radial ridges. The larger articulated plates [7], representing an older individual, have additional large, central superimposed denticles associated with the large marginal denticles, which represent a later growth stage than the one represented by the isolated supragnathal [4]. Further, the mode of plate growth in arthrodires is quite distinct from the oral elements of *R. stellina* which exhibit concentric marginal addition (figure 1*a,b*).

Burrow and colleagues [1] contend with our interpretation of the tissues comprising the gnathal plate of *Romundina*. Their arguments hinge on peculiar and readily falsifiable definitions of what constitutes a tooth and enameloid. A pulp cavity is neither necessary nor sufficient for the identification of teeth since chondrichthyan teeth often lack an open pulp (e.g. figure 1*e–g*) cavity that is present, nonetheless, in their dermal tubercles. Birefringence is a property of optical anisotropic materials; it does not define whether a material comprises crystallites. We drew comparison to the single crystallite enameloid that characterizes primitive chondrichthyan teeth and in which component crystallites are difficult to discern, even in living materials.

Ultimately, our thesis is eminently testable, by applying the same non-invasive methods that we employed, to the known articulated gnathal elements. We predict that they will exhibit the same composition, aboral morphology and mode of growth exhibited by the isolated gnathal plate that we have described. In the interim, the available evidence suggests that primitive gnathostome dentitions were capped with enameloid and lacked developmental independence from the dermal skeleton.

## Supplementary Material

Data description and access

## Data accessibility

Data used in this manuscript are archived at http://dx.doi.org/10.5523/bris.37q0cntawxcq1rkktq3e9mr1p.
