# Supplementary material for: Reply to ‘placoderms and the evolutionary origin of teeth’: Burrow et al. (2016)
Source: Biol Lett. 2016 Sep;12(9):20160526. doi: 10.1098/rsbl.2016.0526 (PMC5046931; doi:10.1098/rsbl.2016.0526)
Supplement: Data description and access [file rsbl20160526supp1.docx]

**Data description and access**

The supragnathal of *Romundina stellina* (NRM-PZ P.15956; Fig. 1*a*-*d*) was already published in our original paper and reconstructed raw slice data are available at the University of Bristol at http://dx.doi.org/10.5523/bris.7h9gynbsui4u1hap471inrlua and as movie files in the electronic supplementary material. Two blocks of a stacked scan are available folders are named “MR07A_B1_” and “MR07A_B2_”. The specimen was scanned using SRXTM at the TOMCAT (X02DA) beamline of the Swiss Light Source Paul Scherrer Institut with a X10 objective resulting voxel size is 0.74 µm. Energy of 13 keV with 1501 projections equi-angularly distributed over 180˚ and exposure time of 175 ms was used.

Two teeth of *Scyliorhiuns canulicula* were extracted from a specimen housed in the collection of the University of Bristol (BRSUG 29402). The reconstructed raw slice data are available at the University of Bristol ([https://data.bris.ac.uk/webshare/glpcjd-donoghue-2012/d43ea957-b0e5-4b19-ae62-dc809b174df4/](https://data.bris.ac.uk/webshare/glpcjd-donoghue-2012/d43ea957-b0e5-4b19-ae62-dc809b174df4/)). Volumetric data sets were produced using SRXTM at the TOMCAT (X02DA) beamline of the Swiss Light Source Paul Scherrer Institut, Switzerland. Data consist of two folders each includes reconstructed tiff files:

SRXTM data are, the first tooth of *Scyliorhiuns canulicula* (BRSUG 29402-1; Fig. 1*e*), in the folder named “MR273b_”. The specimen was scanned with a X10 objective resulting voxel size is 0.65 µm. Energy of 14 keV with 1501 projections equi-angularly distributed over 180˚ and exposure time of 175 ms was used. The second tooth of *Scyliorhiuns canulicula* (BRSUG 29402-2; Fig. 1*f*, *g*) was scanned in higher resolution as region of interest scan using propagation phase contrast, reconstructed tiffs in folder “MR276a” were scanned with X40 objective resulting voxel size of 0.16 µm. Energy of 15 keV with 1501 projections and exposure time of 550 ms was used.

No human or animal experiments have been conducted for this research.
